# Plasma extracellular vesicles-derived microRNAs provide potential biomarkers in distinguishing between focal cortical dysplasia type I and II

**DOI:** 10.1038/s41390-025-04343-z

**Published:** 2025-11-02

**Authors:** Bocheng Zhou, Hao Yu, Chang Liu, Yao Wang, Yi Wang, Ruofan Wang, Qingzhu Liu, Taoyun Ji, Shuang Wang, Huili Wang, Ye Wu, Xiaoyan Liu, Yuwu Jiang, Lixin Cai, Yu Sun

**Affiliations:** 1 Pediatric Epilepsy Center, Peking University First Hospital, Beijing, China; 2 Department of Pediatrics, Peking University First Hospital, Beijing, China; 3 School of General Practice and Continuing Education, Capital Medical University, Beijing, China

## Abstract

**Background:**

Preoperative pathological information of focal cortical dysplasia (FCD) is critical for surgical planning, but non-invasive diagnostic methods are currently unavailable. This study aimed to identify potential biomarkers for FCD subtyping by screening microRNAs (miRNAs) in plasma extracellular vesicles (EVs).

**Methods:**

We identified the most representative pathological subtypes based on the clinical characteristics of FCD Type I and II in 439 pediatric FCD patients. Differential expression analysis of miRNA was performed in plasma EVs and brain tissues from samples of representative pathological subtypes. Potential biomarkers and downstream target genes were identified by integrating brain tissue transcriptome data. Target genes underwent enrichment and protein-protein interaction analyses, with immunohistochemical validation in brain tissue.

**Results:**

FCD Ia and IIb represent the clinical characteristics between FCD Type I and II, respectively. Eight differentially expressed miRNAs common to plasma EVs and brain tissue were identified, which had 241 target genes in brain tissue. These target genes were enriched in immune-related functions, cytokine-cytokine receptor interaction, p53, and NF-κB signaling pathways. Among nine core proteins, CDKN1A and CD274 were confirmed to be elevated in FCD IIb lesions compared to FCD Ia.

**Conclusion:**

Differentially expressed miRNAs in extracellular vesicles may serve as potential non-invasive biomarkers for FCD subtyping.

**Impact:**

- Lesional characteristics, surgical approaches, and prognosis are different between FCD type I and II.
- Eight differentially expressed miRNAs in plasma extracellular vesicles between FCD type I and II were identified.
- Target genes of these miRNAs were significantly enriched in immune and inflammatory responses and cytokine pathways.
- The identified miRNAs may represent potential pre-operative biomarkers for FCD subtyping, potentially guiding surgical strategies and outcome prediction, especially for FCD type II.
- Extracellular vesicle-mediated immune responses may differ between FCD type I and II, offering insight into FCD pathogenesis, epileptogenic mechanisms, and the identification of novel therapeutic targets.

## Introduction

Focal cortical dysplasia (FCD) is the most common cause of drug-resistant epilepsy in pediatric patients undergoing surgery.^1^ FCD can be categorized as type I, type II, type III, mMCD (mild malformations of cellular development), MOGHE (mMCD with oligodendroglial hyperplasia in epilepsy), and no definite FCD on histopathology by the ILAE classification criteria.^2^ FCD type I and type II have the highest proportion in pediatric patients submitted to epilepsy surgery, accounting for 6.4% and 17.0%, respectively.^1^ FCD type I is characterized by architectural disorganization of the neocortex, where Ia has an abundance of neuronal microcolumns and heterotopic neurons in the white matter, Ib shows the disruption of the six-layered anatomical organization, and Ic exhibits both vertical and horizontal structural abnormalities.^2^ FCD type II is an isolated lesion characterized by cortical dyslamination and dysmorphic neurons (DNs) without (type IIa) or with balloon cells (type IIb).^2^

In terms of structure, FCD type I lesions are mainly MRI-negative and more extended than FCD type II,^3,4^ while FCD type II lesions are more localized and display characteristic imaging features, such as the “transmantle” sign.^3,5,6^ Notably, MRI-negative cases approach one-third in FCD type II, complicating preoperative differentiation between the two FCD subtypes.^3,7^ From the aspect of functional network connectivity, FCD type I exhibits higher excitability than type II in the ictal zone and brain regions distant from the ictal zone.^8,9^ The epileptogenic zone and connectivity patterns in type I are larger and more intricate than in type II, indicating that FCD type I tends to form a more extended epileptogenic network than type II.^8,9^ In fact, resection for FCD type I demands greater amounts and extent of brain tissue, and the proportion of patients adopting disconnection surgery is also higher than that of FCD type II surgery.^9,10^ Preoperative pathological subtyping may provide important information for surgical decision-making. However, there are no relatively objective biomarkers to differentiate between FCD type I and type II preoperatively. And the underlying biological mechanism of differences in functional network connectivity is unclear.

Beyond the physical connectivity between brain regions, the formation of extensive epileptogenic networks and the interactions among their constituent nodes may also be modulated by specific humoral factors. Extracellular vesicles (EVs) represent one such potential mediator.^11,12^ Extracellular vesicles (EVs) are lipid-bilayer-containing granules that are released by cells and are incapable of self-replication.^13^ Particular protein, lipid, and nucleic acid molecules are encapsulated in EVs, which are transported by body fluids from one cell to another. This mechanism, which is universally observed in nature, regulates different cellular processes.^14–16^ MicroRNA (miRNA) is a noncoding double-stranded RNA molecule with a length of 20–22 nucleotides, processed from RNA precursors. These small RNA molecules mainly regulate gene expression at the post-transcriptional level by attaching to the 3’-untranslated region (3’-UTR) of the target mRNA.^17^ EVs provide a stable environment for miRNA, which can traverse the blood-brain barrier and blood-cerebrospinal fluid barrier via EVs. This allows miRNA to regulate an extensive spectrum of cell activities and get involved in several neurological disorders, such as epilepsy.^18–20^

Using clinical data from our pediatric epilepsy center, we analyzed the differences between FCD types I and II and the four pathological subtypes in terms of the location and number of lobes involved in the lesions, as well as the surgical method used. The two pathological subtypes with the highest differences were chosen as representative samples to assess the differences in miRNA expression in plasma extracellular vesicles (EVs) between FCD types I and II. In this exploratory study, we aim to discover the differences in biological characteristics between FCD types I and II, which may assist in the screening of potential biomarkers for preoperative diagnosis of FCD types I and II, in addition to further understanding of the pathogenic mechanisms of FCD.

## Methods

### The study population of clinical research

Patients were queried from clinical databases in the Pediatric Epilepsy Center of Peking University First Hospital (PECPUFH) between January 1, 2014, and November 31, 2022. Patients were selected based on the following criteria: (1) Age of operation ≤ 18 years old; (2) Drug-resistant refractory epilepsy according to the criteria defined by the International League Against Epilepsy (ILAE): failure of adequate trials of two tolerated and appropriately chosen and used antiepileptic drug (AED) schedules (whether as monotherapies or in combination) to achieve sustained seizure freedom^21^; (3) Clinicopathologic diagnosis of FCD confirmed by independent pathologist and excluded from “dual pathology” according to the ILAE 2022 classification guidelines.^2^ (4) Follow up at least 3 months after surgery. Exclusion criteria included: (1) pathological diagnosis of FCD associated with hippocampal sclerosis, cerebrovascular diseases, brain tumors, and acquired lesions (including traumatic injury, ischemic injury, or encephalitis); and (2) cases in which no definite pathological diagnosis could be obtained. All research protocols were approved by the institutional review board of the ethics committee of Peking University First Hospital.

### Dysmorphic neuron identification

The pathological categorization for each sample was made according to the ILAE 2022 classification guidelines.^2^ All the pathological reports of the patients were reviewed by two independent investigators. Dysmorphic neurons are exclusively characterized by the following set of severe cytologic abnormalities: (1) Neuronal cell diameters are significantly enlarged, ranging from 16–43 μm compared to 12–25 μm in normal-appearing pyramidal neurons in layer 3; (2) the cell nucleus diameter is also significantly enlarged, ranging from 15–28 μm compared to 10–18 μm in normal pyramidal cells in layer 3; (3) Nissl substance is aggregated and displaced toward the cell membrane; (4) phosphorylated (antibody 2F11) and non-phosphorylated neurofilament isoforms (SMI-32) accumulate in their cytoplasm.^2,22^

### Sequencing sample information and sample collection

**Fig. 1:**
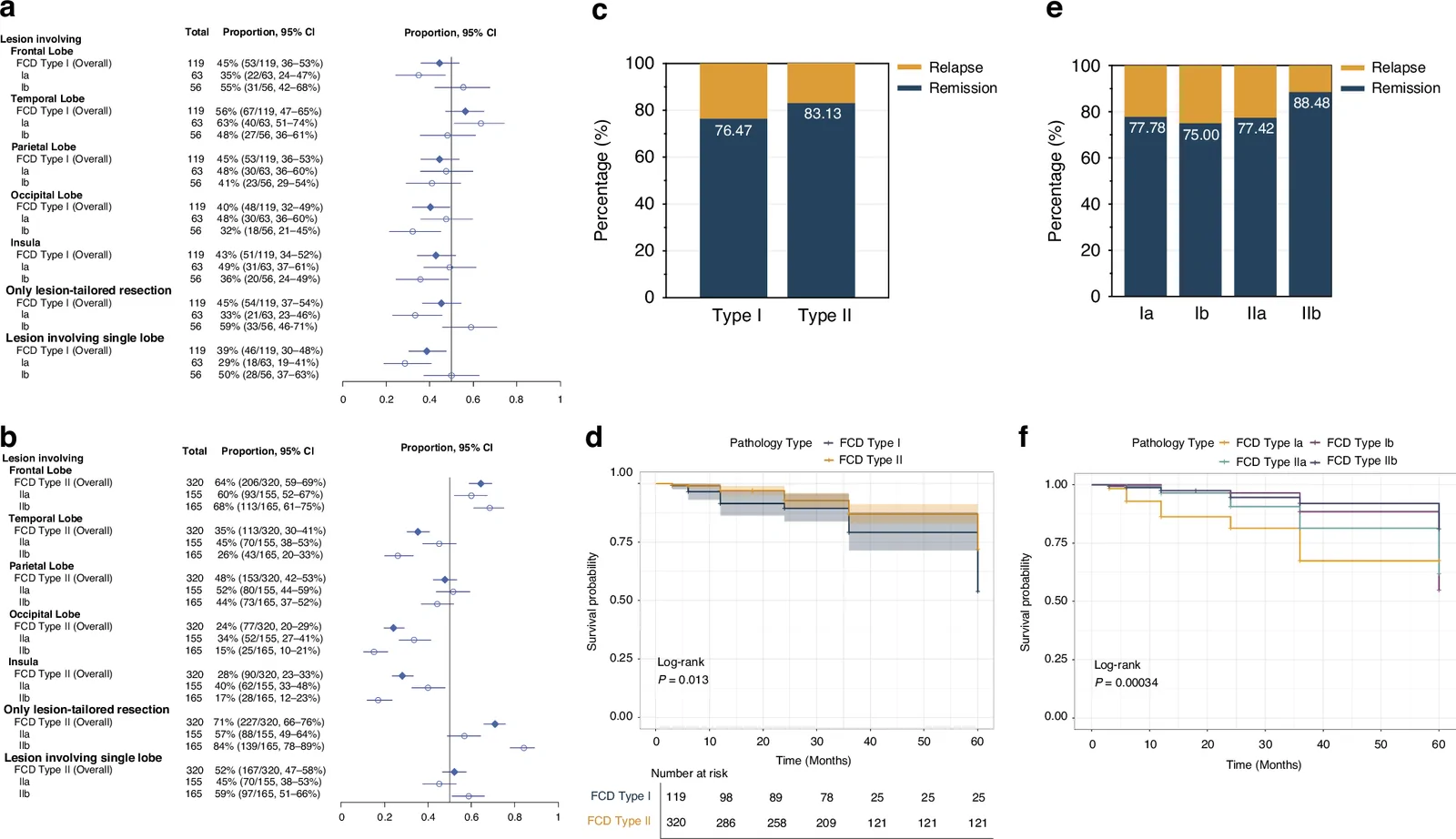
The differences between FCD types I and II in lesion location, extent, surgical approach and prognosis. **a** The proportion of lesion location, extent, and surgical approach in FCD type I and its pathological subtypes. **b** The proportion of lesion location, extent, and surgical approach in FCD type II and its pathological subtypes. **c** Recurrence-remission proportions for FCD type I and type II following surgery. **d** The Kaplan-Meier curve of FCD type I and type II. The difference in surgical prognosis between these two pathology types was statistically significant (*p* < 0.05). **e** Recurrence-remission rates of postoperative seizure in 4 pathological subtypes. **f** The Kaplan-Meier curve of 4 pathological subtypes. The difference in surgical prognosis among these pathological types was statistically significant (*p* < 0.05). Abbreviations: FCD, focal cortical dysplasia.

A total of 10 patients with FCD Ia and 6 patients with FCD IIb were enrolled, who underwent epilepsy surgical treatment from January 1, 2019, to November 31, 2022, in our pediatric epilepsy center. Blood samples (10 mL) were drawn from a central venous catheter, placed in EDTA tubes, gently inverted five or six times to mix, and centrifuged for 10 min at 3000 g and 4 °C. The upper layer (plasma) was aspirated and stored at −80 °C before usage. Brain tissue was surgically resected to treat drug-resistant epilepsy at PECPUFH. Resected tissue was fresh-frozen for subsequent analysis. The flow diagram of sample processing is presented in Fig. 1. All protocols were approved by the Ethics Committee of Peking University First Hospital.

### Isolation and purification of plasma EVs

For each patient, 3 mL of plasma was utilized, and to remove cell debris, the plasma was centrifuged at 3000×g for 15 min after it had been thawed at 37 °C. The supernatant is diluted with seven volumes of phosphate-buffered saline (PBS), centrifuged for thirty minutes at 13,000 × g, and then run through a 0.22 μm filter to get rid of big particles. To pellet the EVs, the supernatant was ultracentrifuged using a P70AT rotor (RP70AT; Hitachi) at 150,000×g (45,200 rpm) and 4 °C for 4 h. To resuspend the pellet, mix it with PBS and centrifuge it at 150,000 × g and 4 °C for 2 h. The EVs-enriched pellet was resuspended in 100 μL of PBS following PBS washing, then stored at −80 °C.

### Transmission electron microscopy (TEM)

Following ultracentrifugation, the EVs were diluted from 5 μL to 10 μL and forwarded for analysis. Take 10 μL of the sample dropwise, make it precipitate on a copper mesh for one minute, and then absorb the float with the filter paper. The copper mesh received 10 mL of phosphotungstic acid dropwise for 1 minute, and the filter paper absorbed the float. For a few minutes, leave it to dry at room temperature. 80 kV for imaging using electron microscopy.

### Nanoparticle tracking analysis (NTA)

The EVs sample was diluted to 30 μL by 5 μL and passed on for analysis. Before loading the EVs sample, the instrument performance test with the standard is utilized. The NanoFCM device (Flow NanoAnalyzer) could be used to determine the particle size and concentration once the sample has been evaluated.

### Western blot (WB)

The EV-enriched fractions were denatured in 5× sodium dodecyl sulfonate (SDS) buffer and subjected to western blot analysis (10% SDS-polyacrylamide gel electrophoresis; 50 μg protein/lane) using rabbit polyclonal antibody TSG101 (Santa Cruz, sc-13,611), syntenin (Abcam, ab133267), and calnexin (Abcam, ab22595). The membranes were visualized on a ChemiDoc MP Imager (BioRad) following development with Clarity Western Enhanced Chemiluminescence Blotting Substrate (BioRad).

### Immunohistochemistry (IHC)

Immunohistochemistry was performed on 4 µm sections from formalin-fixed, paraffin-embedded (FFPE) focal cortical dysplasia (FCD) tissues. Following deparaffinization and rehydration, antigen retrieval was conducted by heating sections in a pressure cooker for 2 min, using citrate buffer (pH 6.0; G1201, Servicebio) for CDKN1A detection or TE buffer (pH 9.0; G1203, Servicebio) for CD274 detection. Endogenous peroxidase activity was quenched with 3% H_2_O_2_, and non-specific sites were blocked with 3% BSA. Sections were then incubated overnight at 4 °C with primary antibodies: recombinant anti-p21 mouse monoclonal antibody (targeting CDKN1A, 1:1000; GB15153, Servicebio) and anti-PD-L1 rabbit polyclonal antibody (targeting CD274, 1:300; GB115736, Servicebio). Subsequently, sections were incubated with HRP Goat Anti-Mouse IgG (1:200; GB23301, Servicebio) for 1 hour at room temperature for CDKN1A detection or with S-vision poly-HRP conjugated Goat Anti-Rabbit IgG (H + L) (G1302, Servicebio) for 20 min at room temperature for CD274 detection. Immunoreactivity was visualized using 3,3’-diaminobenzidine (DAB), and sections were counterstained with hematoxylin, dehydrated, cleared, coverslipped, and imaged using a white light microscope.

### RNA isolation

Total RNA was extracted and purified from EV samples using the miRNeasy Mini kit (Qiagen, cat. No. 217,004). Each EV sample was mixed with 700 μL of QIAzol Lysis Reagent vigorously with a vortex and incubated for 5 min at RT. Then 100 μL of chloroform was added and mixed vigorously for 15 s, followed by a 2 min incubation at RT. Separation of the aqueous phase that contained RNA from the organic phase occurred in a centrifuge at 12,000 × g, 4 °C for 15 min. The aqueous phase was transferred into a new 1.5 mL tube, and 2 volumes of 100% ethanol were added to the tube and mixed with pipetting up and down. This ethanol mixture was loaded in the volume of 700 μL into the RNeasy MinElute Column (Qiagen) and spun down at 9000 × g for 30 s at RT. The column loading and spinning were repeated with the remaining ethanol mixture. The column was washed with 500 μL of RWT buffer (initially prepared in 100% isopropanol) and spun down at 9000 × g for 30 s at RT. The other 2 washing steps used 500 μL of RPE buffer (initially prepared in 100% ethanol) and the same spinning condition. Additional spinning at full speed for 3 min was performed to dry the membrane in the column. The RNA elution step was undertaken with 30 μL of nuclease-free water that was applied to the membrane, followed by incubation for 2 min at RT and spinning at 10,000 × g for 1 min.

### Library preparation and RNA-Seq for transcriptome

RNA purification, reverse transcription, library construction, and sequencing were performed at Shanghai Majorbio Bio-pharm Biotechnology Co., Ltd. (Shanghai, China) according to the manufacturer’s instructions (Illumina, San Diego, CA). The brain tissue RNA-seq transcriptome library was prepared following Illumina Stranded mRNA Prep, Ligation from Illumina (San Diego, CA) using 1 μg of total RNA. Shortly, messenger RNA was isolated according to the polyA selection method by oligo (dT) beads and then fragmented by fragmentation buffer first. Secondly, double-stranded cDNA was synthesized using a SuperScript double-stranded cDNA synthesis kit (Invitrogen, CA) with random hexamer primers (Illumina). Then the synthesized cDNA was subjected to end repair, phosphorylation, and ‘A’ base addition according to Illumina’s library construction protocol. Libraries were size-selected for cDNA target fragments of 300 bp on 2% Low Range Ultra Agarose, followed by PCR amplification using Phusion DNA polymerase (NEB) for 15 PCR cycles. After being quantified by Qubit 4.0, the paired-end RNA-seq sequencing library was sequenced with the NovaSeq Xplus sequencer (2 × 150 bp read length). The raw paired-end reads were trimmed, and quality was controlled by fastp^23^ with default parameters. Then clean reads were separately aligned to the reference genome with orientation mode using HISAT2^24^ software. The mapped reads of each sample were assembled by StringTie^25^ in a reference-based approach.

### Library preparation and RNA-Seq for miRNA

RNA purification, reverse transcription, library construction, and sequencing were performed at Shanghai Majorbio Bio-pharm Biotechnology Co., Ltd. (Shanghai, China) according to the manufacturer’s instructions (Illumina, San Diego, CA). Using high-throughput sequencing platforms, sequence the enriched small RNA fragments of 16–35 nt. The library is constructed using the SMARTer smRNA-Seq Kit (Illumina, San Diego, CA) with 10 ng of total RNA, and the operation process is as follows. To facilitate cDNA synthesis using oligo(dT) primers, treat miRNA with poly(A) polymerase to add a poly(A) tail. 3’ adapter primers are ligated, and the RNA with the ligated adapters is used as a template to synthesize the first-strand cDNA via reverse transcription. Then, 5’ adapter primers are ligated, and template switching occurs to synthesize the complete first-strand cDNA, followed by the synthesis of the second strand. Amplify the library using sequencing primers through PCR (11-12 cycles) to enrich the library concentration. After quantification by Qubit 4.0 and mixing according to the data ratio for sequencing, bridge PCR amplification was performed to generate clusters, and the sequencing library was sequenced on the Illumina NovaSeq Xplus platform. Raw data (raw reads) in FASTQ format were firstly processed through fastp^23^ with default parameters. After this step, clean data (clean reads) were obtained by removing the 3’ end adapter, reads containing poly-N, low-quality bases (Sanger base quality of <20) of the 3’ end, and sequencing adapters from raw data with the fastx toolkit software. All identical sequences of sizes ranging from 18 to 32 nt were counted and eliminated from the initial data set. The mapped small RNA tags were first used to identify known miRNA with the miRBaseV2 database (http://www.mirbase.org/) as a reference. Then the rest of the tags were aligned with the Rfam database and Repbase database to remove ribosomal RNA (rRNA), transfer RNA (tRNA), small nuclear RNA (snRNA), small nucleolar RNA (snoRNA), and other ncRNA and repeats. The unannotated tags were predicted to be and identified as novel miRNAs using mirdeep2.^26^

### Determining representative pathological subtypes

To identify the pathological subtype most representative of the clinical characteristics of its parent major pathological class (FCD Type I or FCD Type II), we employed a quantitative strategy based on clinical feature distribution. This study included a panel of *K* dichotomous clinical variables (coded 0 or 1), encompassing lesion location (frontal, temporal, parietal, occipital, and insular lobes), surgical approach (only lesion-tailored resection), and lesion extension (lesion involving a single lobe). For each major pathological class *M* and each of its constituent subtypes *S*_*j*,_ we first calculated the proportion of patients exhibiting each clinical variable *V*_*k*_ (i.e., $P\left({V}_{k}=1{|M}\right)$ for the major class and $P\left({V}_{k}=1|{S}_{j},M\right)$ for the subtype). The dissimilarity in a single clinical variable *V*_*k*_ between subtype *S*_*j*_ and its parent class *M* was quantified as follows:

$$D\left({S}_{j},{V}_{k},M\right)={\left[P\left.\left({V}_{k}=1\right){S}_{j},M\right)-P\left({V}_{k}=1{|M}\right)\right]}^{2}$$

A total dissimilarity score (TDS) for each subtype *S*_*j*_ relative to its parent class *M* was then computed by summing these individual dissimilarities across all *K* clinical variables:

$${{\rm{TDS}}}\left({Sj}|M\right)={\sum}_{k=1}^{K}D({S}_{j}{V}_{k}M)$$

The pathological subtype ${S}_{j}^{* }$ yielding the minimum TDS within its major class *M* was designated as the most representative of that major class’s clinical profile. All analyses were performed using R 4.0.3.

### Bioinformatic and statistical analysis

The Wilcoxon rank sum test, Fisher’s exact test, and Pearson’s chi-squared test were applied in R 4.0.3 to compare clinical data between groups. Kaplan-Meier survival curves were constructed to visually depict the prognosis of the different groups, and the rate of difference in the occurrence of events was compared using the log-rank method. Multiple comparisons were performed using the Benjamini-Hochberg method (BH). *p* < 0.05 was considered statistically significant. To identify differentially expressed genes (DEGs) between two different samples, the expression level of each transcript was calculated according to the read counts. Differential expression analysis was performed using the DESeq2.^27^ DEGs or differential expression miRNAs (DEMs) with |log2(Fold Change)| ≧ log2(1.5) and *p* <0.05 were considered to be significantly differently expressed. The multiMiR package^28^ was used to predict the target genes for the potential miRNAs, and brain tissue DEGs were cross-referenced with the results. Functional enrichment analyses, including GO (Gene Ontology) and KEGG (Kyoto Encyclopedia of Genes and Genomes), were carried out in R 4.0.3. Protein-protein interaction (PPI) networks were constructed using the STRING database (version 12.0, https://string-db.org) to analyze potential functional associations between target genes. The interaction data were analyzed and visualized in Cytoscape (version 3.10.1). Network nodes were ranked and visualized according to their degree centrality, a key graph-theoretical measure reflecting the number of direct connections for each protein.

## Results

### FCD Ia and FCD IIb represent the characteristics of FCD I and II in lesion location, extension, surgical approach, and outcomes

Out of the 439 participants enrolled, there were 119 FCD type I patients (Ia = 63, Ib = 56) and 320 FCD type II patients (IIa = 155, IIb = 165). The demographic and clinical information was summarized in Table 1 and Supplementary Table S1. Regarding lesion location, FCD type I lesions predominantly involved the temporal lobe, while FCD type II lesions more frequently affected the frontal lobe. Notably, FCD type I lesions demonstrate a significantly higher incidence of involving the occipital and insular lobes compared to FCD type II (Table 1, Fig. 1A, B). Based on the number of affected lobes, FCD type I lesions exhibit more extensive involvement than FCD type II (Table 1, Fig. 1A, B). This finding is consistent with the observation that dissecting surgery is typically performed for FCD type I, whereas lesion-tailored resection is more commonly chosen for FCD type II (Table 1, Fig. 1A, B). FCD Ia and FCD IIb display the lowest total dissimilarity score (TDS) within FCD type I and type II, respectively. These two subtypes consequently exhibit minimal deviation from their parent types across all three clinical characteristics (Table 2).

**Table 1:** The demographic and surgical information of FCD type I and II

Characteristic	Overall (*N* = 439)	FCD Type I (*N* = 119)	FCD Type II (*N* = 320)	*p*-value
Gender				0.5
Male	269 (61.28%)	76 (63.87%)	193 (60.31%)	
Female	170 (38.72%)	43 (36.13%)	127 (39.69%)	
Seizure onset age (Days)	307 (96.50, 1009)	306 (97.00, 998)	315 (97.25, 1007)	0.7
Surgery age (Days)	1503 (850.00, 2492)	1589 (945.50, 2567)	1438 (800.25, 2486)	0.14
Frequency of seizures >10 times per day	156 (35.54%)	39 (32.77%)	117 (36.56%)	0.5
Surgery side				0.2
Left	228 (51.94%)	68 (57.14%)	160 (50.00%)	
Right	211 (48.06%)	51 (42.86%)	160 (50.00%)	
Lesion involving				
Frontal lobe	259 (59.00%)	53 (44.54%)	206 (64.38%)	<0.001
Temporal lobe	180 (41.00%)	67 (56.30%)	113 (35.31%)	<0.001
Parietal lobe	206 (46.92%)	53 (44.54%)	153 (47.81%)	0.5
Occipital lobe	125 (28.47%)	48 (40.34%)	77 (24.06%)	<0.001
Insula	141 (32.12%)	51 (42.86%)	90 (28.13%)	0.003
Lesion involving single lobe	213 (48.52%)	46 (38.66%)	167 (52.19%)	0.012
Lesion involving multiple lobes	226 (51.48%)	73 (61.34%)	153 (47.81%)	0.012
The number of lobes involved				0.011
1	213 (48.52%)	46 (38.66%)	167 (52.19%)	
2	111 (25.28%)	33 (27.73%)	78 (24.38%)	
3	25 (5.69%)	9 (7.56%)	16 (5.00%)	
4	49 (11.16%)	22 (18.49%)	27 (8.44%)	
5	41 (9.34%)	9 (7.56%)	32 (10.00%)	
Disconnection surgery	158 (35.99%)	65 (54.62%)	93 (29.06%)	<0.001
Only lesion-tailored resection	281 (64.01%)	54 (45.38%)	227 (70.94%)	<0.001

*Median (IQR); n (%).

*FCD* focal cortical dysplasia.

**Table 2:** Total Dissimilarity Score for each pathological subtypes

Pathological Type		TDS
FCD Type I	Ia	0.0493792
	Ib	0.0624956
FCD Type II	IIa	0.0611547
	IIb	0.0539666

*FCD* focal cortical dysplasia, *TDS* Total Dissimilarity Score.

The surgical prognosis of FCD type II was better than FCD type I, with a seizure-free rate of above 80% after surgery (Fig. 1C, D). There were differences in the prognosis of the four pathological subtypes of FCD types I and II (Fig. 1F), with the best surgical prognosis (88.48%) for FCD IIb (Fig. 1E). The surgical prognosis of FCD Ia, Ib, and IIa was not significantly different (Supplementary Fig. S1A, B, D), while FCD IIb and FCD Ia showed the greatest variation in prognosis (Supplementary Fig. S1C).

**Fig. 2:**
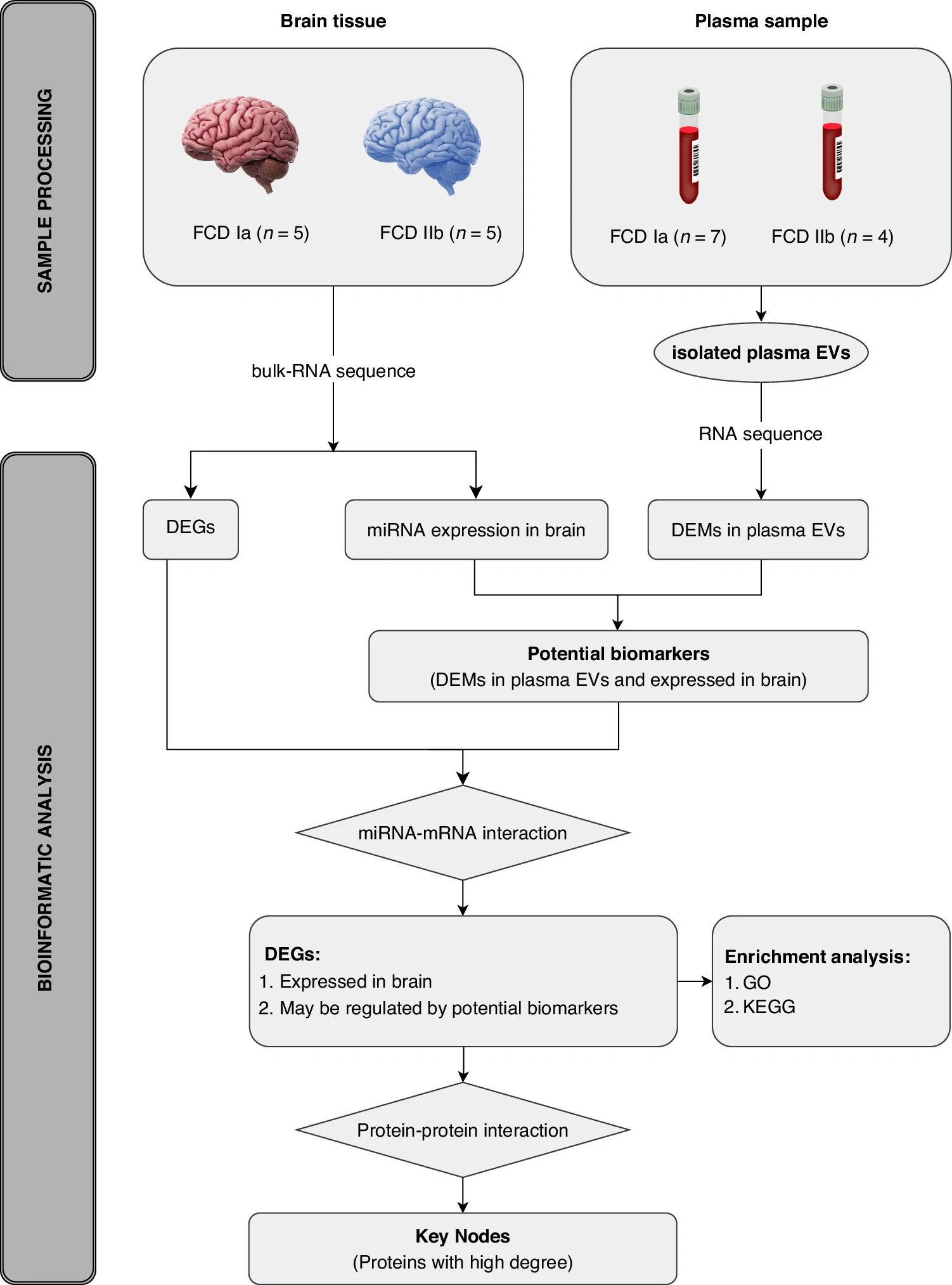
The sample processing and bioinformatics analysis process in this study. FCD focal cortical dysplasia, EVs extracellular vesicles, DEGs differential expression genes, DEMs differential expression miRNAs, GO Gene Ontology, KEGG Kyoto Encyclopedia of Genes and Genomes.

These findings indicate that the variations between FCD Ia and IIb in lesion location, extension, surgical approach, and surgical prognosis represented the differences between FCD types I and II. Therefore, these two pathological subtypes were selected for further analysis (Fig. 2).

### Isolation and characterization of EVs from plasma

**Fig. 3:**
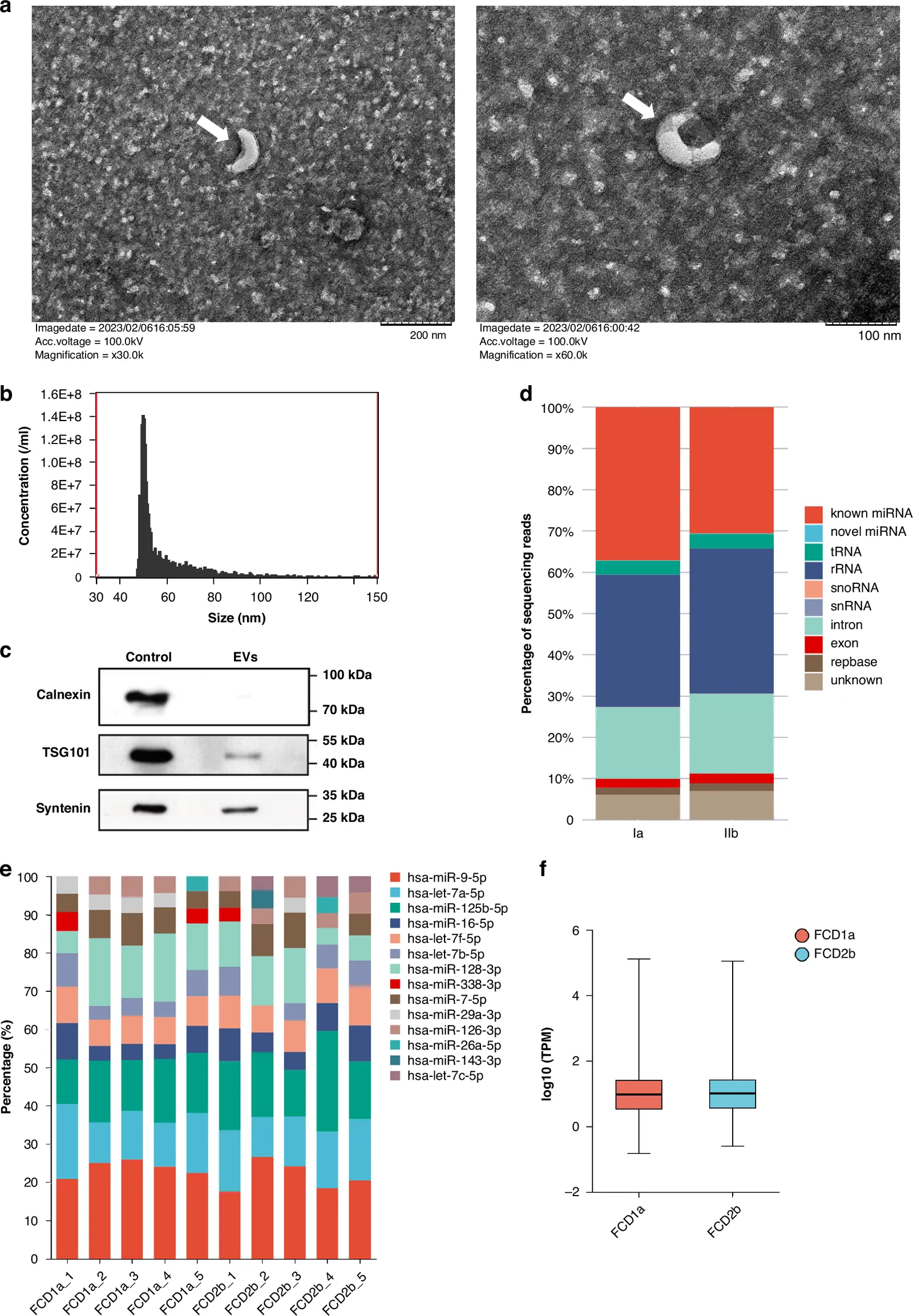
Characterization of EVs from participant plasma and RNA expression profile in brain tissue. **a** TEM images showed EVs to be nucleus-free oval or bowl-shaped capsules. **b** NTA results indicated that the diameter of EVs enriched from plasma was around 30-150 nm. **c** EV markers TSG101 and Syntenin were all identified in EV-enriched fractions isolated from plasma, however, Calnexin, a negative EV marker, was not found in EV samples. **d** The bar plot comparing the small RNA species of EVs enriched fractions isolated from FCD Ia and IIb groups. **e** The bar plot comparing the top 10 miRNA in brain tissue from FCD Ia and IIb groups. (**f**) The box plot revealed no statistically significant difference in brain tissue gene expression level between the FCD Ia and IIb groups. FCD focal cortical dysplasia, EVs extracellular vesicles, TEM Transmission electron microscopy, NTA Nanoparticle tracking analysis.

In this study, plasma EVs were isolated from FCD Ia (*n* = 7) and FCD IIb (*n* = 4). The demographic and clinical information of the participants, including age, sex, resection site, and other relevant details, were presented in Table 3. The plasma EVs were extracted from the plasma of 11 FCD patients by ultracentrifugation, and the morphology and size distribution of the EVs were evaluated by TEM and NTA. The TEM and NTA analysis showed that EVs present in the isolated fractions had an oval or bowl-shaped morphology, with a size ranging from 30 nm to 150 nm (Fig. 3A, B). The EV markers Syntenin and TSG101 were both shown to be abundant in the EVs isolated from the plasma (Fig. 3C). In contrast, our isolated EV-enriched fraction samples did not include Calnexin, which is a negative sign of EVs (Fig. 3C).

**Table 3:** The clinical demographics of plasma EVs and brain tissue

Case	Pathology Type	Gender	Seizure onset age (Days)	Surgery age (Days)	Surgery side	Plasma EVs extraction	Plasma EVs miRNA sequence	Brain tissue and sequence
1	Ia	Male	820	2447	R	Y	N	Y
2	Ia	Male	232	1983	L	Y	Y	N
3	Ia	Male	547	1569	R	Y	N	Y
4	Ia	Male	396	1499	R	Y	Y	Y
5	Ia	Male	220	831	L	Y	Y	N
6	Ia	Female	186	1733	L	Y	Y	N
7	Ia	Female	1096	2481	R	Y	N	Y
8	Ia	Female	106	473	L	Y	Y	N
9	Ia	Male	584	1047	R	Y	Y	N
10	Ia	Female	891	1324	L	Y	Y	Y
11	IIb	Male	247	902	L	Y	Y	Y
12	IIb	Female	1277	1995	L	Y	N	Y
13	IIb	Female	1004	1469	R	Y	Y	Y
14	IIb	Male	918	1497	R	Y	Y	Y
15	IIb	Female	259	747	R	Y	Y	N
16	IIb	Male	1495	3417	L	N	N	Y

*FCD* focal cortical dysplasia, *EVs* extracellular vesicles.

### Identification of potential miRNAs and target genes related to FCD Ia and IIb

The analysis and quantification of RNA extracted from both EV-enriched fractions and brain tissue were performed. No significant difference was identified in small RNA species of EVs between FCD Ia and IIb (Fig. 3D). Most miRNAs and the overall expression level of the gene were found to be common between both FCD Ia and IIb in brain tissue (Fig. 3E, F).

**Fig. 4:**
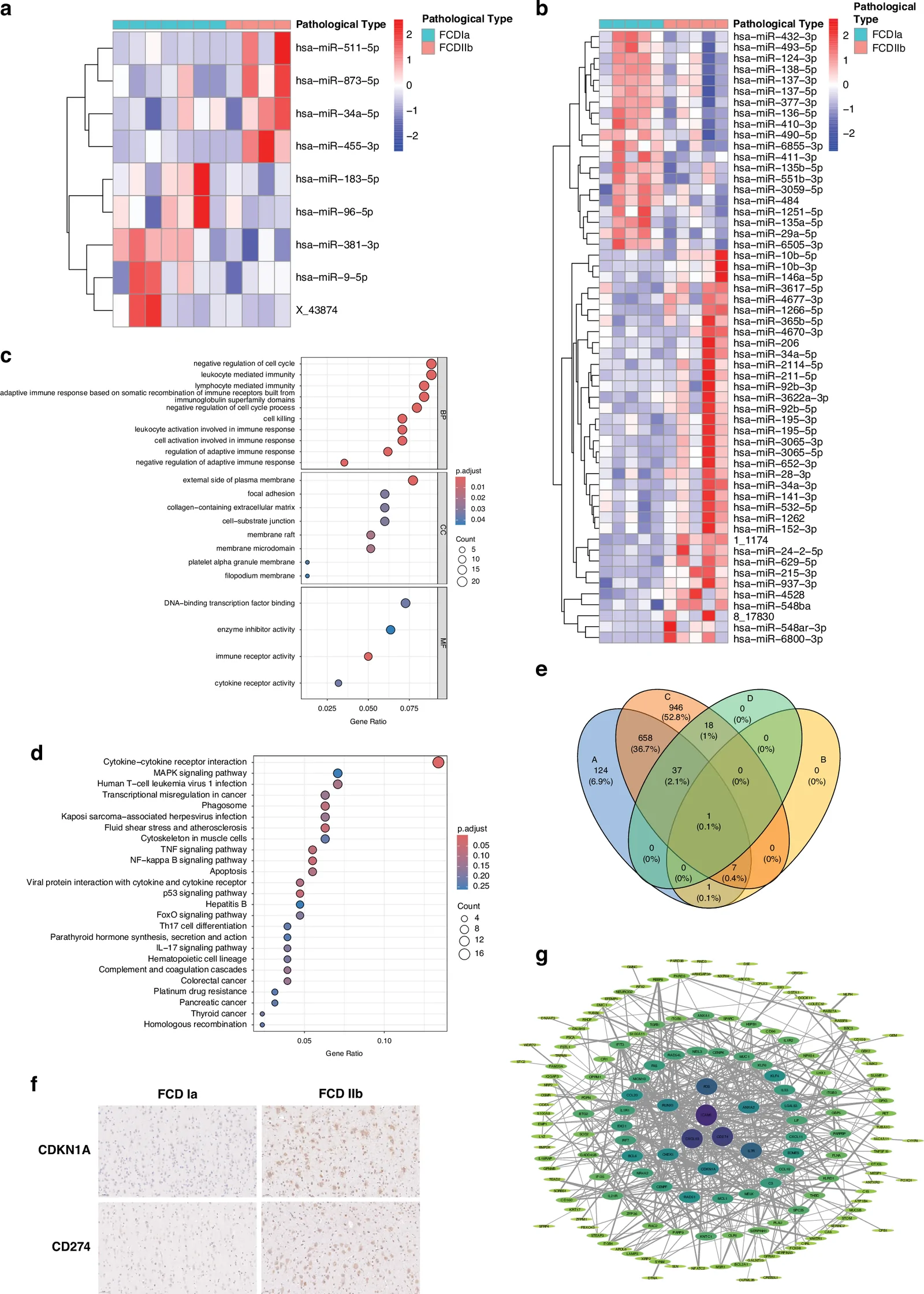
miRNAs profiles and target analysis of differentially expressed miRNAs in plasma EVs of FCD Ia and IIb groups. **a** The heatmap of differently expressed miRNA in plasma EVs of FCD Ia and IIb. hsa-miR-34a-5p, hsa-miR-455-3p, hsa-miR-873-5p, and hsa-miR-511-5p are the four miRNAs that showed high expression in FCD IIb. FCD Ia showed high expression of five miRNAs: hsa-miR-96-5p, hsa-miR-9-5p, hsa-miR-183-5p, hsa-miR-381-3p, and X_438/4. Except for X_438/4, all miRNAs were known. **b** The heatmap of differently expressed miRNA in brain tissue of FCD Ia and IIb. There are 20 miRNAs highly expressed in FCD IIb and 36 miRNAs highly expressed in FCD Ia. All are known miRNAs except 1_1174 and 8_17830. **c** The bubble plot of GOs enriched, including Biological Process (BP), Cellular Component (CC), and Molecular Function (MF). Target genes were selected from the database to identify those that were differentially expressed in the brain tissue of FCD Ia and IIb groups and that interacted with the eight DEMs in plasma EVs. **d** The bubble plot of KEGG pathways enriched. **e** The Venn plot showed the relationship between miRNA expression in plasma EVs and brain tissue. Circle A represents miRNAs expressed in plasma EVs; circle B represents DEMs in plasma EVs of FCD Ia and IIb; circle C represents miRNAs expressed in brain tissue; and circle D represents DEMs in brain tissue of FCD Ia and IIb. 828 miRNAs were identified in plasma EVs, and 703 miRNAs were expressed in brain tissue, 8 of which were differently expressed in plasma EVs. hsa-miR-34a-5p is differentially expressed in both plasma EVs and brain tissue of FCD Ia and IIb. **f** Immunohistochemistry of CDKN1A and CD274 in FCD lesions. These two immunoregulatory proteins exhibited strong positivity in FCD IIb but lower expression in FCD Ia. **g** Protein-protein interaction network analysis of downstream target genes, ranked and visualized based on degree centrality. The core network comprised nine hub proteins, with ICAM1 displaying the highest degree, followed by CD274, CXCL10, FOS, IL7R, ANXA2, CDKN1A, RUNX1, and CHEK1. Abbreviations: FCD, focal cortical dysplasia; EVs, extracellular vesicles; DEMs, differential expression miRNAs; GO, Gene Ontology; KEGG, Kyoto Encyclopedia of Genes and Genomes.

The miRNAs in the plasma EVs of 11 FCD patients were sequenced, and the read counts were used for difference analysis by DESeq2. There were 9 differentially expressed miRNAs (DEMs) found to be expressed differently in FCD Ia and IIb (Fig. 4A). Four miRNAs were up-regulated in FCD IIb compared to FCD Ia, which were hsa-miR-34a-5p, hsa-miR-455-3p, hsa-miR-873-5p, and hsa-miR-511-5p. And five miRNAs were down-regulated in FCD IIb compared to FCD Ia, which were hsa-miR-96-5p, hsa-miR-9-5p, hsa-miR-183-5p, hsa-miR-381-3p, and X_438/4. The heatmap of these DEMs is shown in Fig. 4A. All miRNAs were known except for X_438/4, which was a novel miRNA. A total of 56 DEMs were identified in the bulk-miRNA sequence of brain tissue (Fig. 4B). Compared to FCD Ia, FCD IIb had 20 up-regulated miRNAs and 36 down-regulated miRNAs. All of them were known miRNAs except for 1_1174 and 8_17830, which were novel miRNAs. In the transcriptome sequence of brain tissue, 422 genes were up-regulated and 161 genes were down-regulated in FCD IIb compared to FCD Ia (Supplementary Fig. S2).

A total of 828 miRNAs were identified in plasma EVs, out of which 703 miRNAs were found to be expressed in brain tissue. There were nine DEMs in plasma EVs for the two pathology types, and all eight of them, except X_438/4, were also expressed in brain tissue. Additionally, hsa-miR-34a-5p exhibited differential expression in FCD Ia and IIb brain tissue. The relationship of these miRNAs is shown in Fig. 4E. The 8 plasma extracellular vesicular DEMs that were also expressed in brain tissue have the potential to serve as biomarkers.

We utilized the multiMiR package to find the regulatory relationships between the above 8 DEMs and 583 DEGs in brain tissue, and 421 miRNA-mRNA pairs were found (Supplementary Table S2). After removing the duplication, a total of 241 target genes were identified (Supplementary Table S3), followed by GO and KEGG analysis.

### Functional enrichment analysis of target genes

The GO analysis comprised three levels: Biological Process (BP), Cellular Component (CC), and Molecular Function (MF) (Fig. 4C and Supplementary Table S4). At the BP level, target genes were primarily enriched in immune-related functions, including the regulation of adaptive immune response, adaptive immune response based on somatic recombination of immune receptors constructed from immunoglobulin superfamily domains, lymphocyte-mediated immunity, and the involvement of leukocytes in immune response, etc. In addition, target genes were also enriched in functions linked to cell cycle and cell stress, such as negative regulation of cell cycle, positive regulation of response to external stimulus, and negative regulation of mitotic cell cycle, etc. At the CC level, target genes were mainly enriched in various membrane structures, including the external side of the plasma membrane, membrane raft, membrane microdomain, etc. The focal adhesion, cell-substrate junction, and collagen-containing extracellular matrix were also included. At the MF level, there was an enrichment of target genes related to immune receptor activity, cytokine binding, cytokine receptor activity, and extracellular matrix binding.

In the KEGG pathway analysis, target genes were enriched in 217 pathways (Supplementary Table S5). The pathways that showed enrichment for the highest number of genes were shown in Fig. 4D. There were 4 pathways whose adjusted *p*-value (*p*.adjust) was less than 0.05. In ascending order of *p*.adjust values, there were Cytokine-cytokine receptor interaction, p53 signaling pathway, NF-kappa B signaling pathway, and Fluid shear stress and atherosclerosis. Among all the pathways, the “Cytokine-cytokine receptor interaction” exhibited the largest number of genes.

### Protein-protein interaction networks and preliminary validation of key nodes

A protein-protein interaction network was constructed from the 241 target genes using the STRING database. Following removal of non-interacting proteins, 175 proteins remained in the network (Supplementary Table S6). Network topology was analyzed in Cytoscape using degree-based ranking (Fig. 4G), identifying nine hub proteins. ICAM1 demonstrated the highest connectivity, followed by CD274, CXCL10, FOS, IL7R, ANXA2, CDKN1A, RUNX1, and CHEK1 in descending order of degree (Supplementary Table S7). CDKN1A and CD274 were markedly elevated in FCD IIb lesions compared to FCD Ia, indicating differential regulation of these immunomodulatory proteins across FCD subtypes (Fig. 4F).

## Discussion

### Clinical value and preliminary exploration of liquid biopsy in identifying FCD type IIb

Surgical intervention is an effective method for treating children with drug-resistant epilepsy caused by FCD, with long-term seizure-free rates varying significantly among different centers, ranging from 47% to 79%.^29^ Multiple risk factors contribute to the suboptimal outcomes of FCD surgery, among which the specific pathological subtypes of FCD and the extent of lesion resection are highly correlated with surgical prognosis.^30^ This study, through a 5-year follow-up of a large-scale single-center cohort of FCD cases, found that the extent of surgical resection/disconnection was significantly greater in FCD Ia cases compared to FCD IIb, consistent with previous studies.^9,10^ Striking a balance between “complete lesion removal” and “preservation of brain functionality” is the core principle ensuring good surgical outcomes in FCD-related epilepsy. Overall, lesions in FCD II, represented by IIb, are more distinct and have a more localized epileptogenic network, whereas those in FCD I, represented by Ia, are more diffuse and subtle, with a more complex epileptogenic network. Surgical strategies for IIb lesions should ideally involve limited “tailored resections” to preserve crucial functions, while Ia lesions might require extensive removal of MRI-negative epileptogenic sites to achieve seizure freedom. However, in the pre-operative evaluation phase, besides imaging techniques, there is still a lack of an objective and effective diagnostic marker that could provide pathological information about the epileptogenic focus to guide personalized surgical strategies. This study identified 8 DEMs in plasma extracellular vesicles and brain tissue samples from patients with FCD Ia and IIb types through sequencing, suggesting these DEMs as potential biomarkers for preoperative pathological typing in FCD. This pre-operative liquid biopsy technique could assist surgeons in making personalized surgical strategies and predicting outcomes, especially for FCD IIb.

### Fluid regulation mechanisms in epileptogenic networks

Studies have found that even minor structural lesions can manifest as widespread functional abnormalities,^31^ and in U-fibers in FCD I and II, scattered neurons are related to excitatory circuits in the cortex and contribute to the formation of FCD epileptogenic networks.^32^ However, these physical anatomical structures alone do not fully explain the state regulation between nodes in epileptogenic networks, such as excitability. The maintenance of normal neuronal physiological functions relies not only on the stability of connection structures but also on the homeostasis of the neuronal microenvironment, where astrocytes,^33^ microglia,^34,35^ and extracellular fluid components^36^ play crucial roles. In larger epileptogenic networks, the functional connections between nodes must satisfy two conditions: first, they must be transportable via blood or other extracellular fluids and maintain stability; second, they must be able to cross the blood-brain and blood-cerebrospinal fluid barriers. Extracellular vesicles serve as a form of intercellular communication and can meet these conditions. Although no studies have directly confirmed the hypothesis that “extracellular vesicles are involved in the formation and regulation of epileptogenic networks,” a substantial body of research has demonstrated their involvement in the pathogenesis of epilepsy.^37–40^ This study, through joint analysis of differentially expressed miRNA and mRNA in FCD patient plasma EVs and surgically removed brain FCD samples, analyzed the regulatory networks between brain tissue miRNA-mRNA and plasma EVs miRNA, proposing new insights into the differences in fluid-brain interactions between FCD Ia and IIb patients. In the study of brain epileptogenic networks, besides the regulatory modes between neural connections, is there participation of fluid regulation mechanisms? This research observed that miRNAs in FCD patient plasma EVs have the potential to regulate genes involved in neural development and neuroimmunity, providing a preliminary theoretical basis for the fluid regulation theory of human brain networks. However, the source of the FCD miRNA and mRNA analyzed in this study is derived from brain tissue, not from extracellular vesicles in the brain tissue interstitial space, thus providing only indirect evidence for their origin from brain tissue. Moreover, this study lacks low-throughput verification of sequencing data and functional validation of differentially expressed miRNA-mRNA interactions, relying only on software predictions for molecular interaction. Further molecular biological validation is needed to verify the experimental results.

### Differential epileptogenic mechanisms in FCD Ia and IIb

Combining transcriptome data from bulk brain tissue RNA-seq and miRNA target gene predictions, we identified miRNA target genes differentially expressed in brain tissue. These target genes are likely regulated by miRNAs in extracellular vesicles, playing distinct roles in the pathogenesis of FCD Ia and IIb. Gene function enrichment analysis revealed that these target mRNAs are significantly enriched in immune and inflammatory responses and cytokine pathways. This finding was further validated by protein-protein interaction (PPI) analysis and immunohistochemistry, suggesting notable differences in immune responses between FCD Ia and IIb. A multicenter multi-omics study also corroborated these results, revealing that somatic variants in focal cortical dysplasia (FCD) type IIb brain tissue demonstrated positive associations with the mTOR pathway and immune-related Gene Ontology (GO) terms, whereas FCD type I exhibited strong positive associations for glycosylation-related processes.^41^ Several factors contribute to these differences. First, activation of the mTOR pathway. Germline mutations, somatic mutations, or “second hits” (germline mutation plus somatic mutation) in mTOR pathway genes can lead to FCD II, megalencephaly (including classic hemimegalencephaly), and tuberous sclerosis complex (TSC).^42^ The mTOR signaling pathway plays a critical role in immune and inflammatory responses,^43^ and abnormal activation of mTOR signaling leads to elevated inflammatory cytokines, a result confirmed in both TSC^44^ and FCD II.^45^ Second, balloon cells are key drivers of inflammatory responses. Studies on brain tissues from FCD IIa and IIb patients surgically removed and compared with autopsy controls found stronger inherent immune, adaptive immune, and cytokine production in FCD IIb, particularly in complement activation and antigen presentation, closely associated with the load of balloon cells in FCD IIb.^46^ Additionally, in KEGG enrichment analysis, target genes were also enriched in some viral infection pathways, such as infection by human T-cell leukemia virus type 1, Kaposi’s sarcoma-associated herpesvirus (human herpesvirus 8, HHV-8), and interactions between viral proteins and cytokine-cytokine receptor pathways. Studies in surgically removed specimens from patients with mesial temporal lobe epilepsy have detected infections of HHV-6 (human herpesvirus 6), herpes simplex virus type 1, and HHV-8.^47^ Similar evidence of viral infections has also been reported in surgically removed specimens from patients with FCD IIb, including the detection of human papillomavirus (HPV) oncoproteins E6 and E7 in FCD IIb, and transfection of E6 and E7 oncoproteins into fetal mouse brains via uterine electroporation causes FCD.^48^ These results suggest that pathogen (e.g., viral) infections may cause cortical developmental abnormalities and induce immune-inflammatory responses.

### Innovation and future work

This study, selecting the clinically most distinct FCD type Ia and IIb, first demonstrated the differential expression of DEMs in extracellular vesicles between these two pathological subtypes, which could serve as potential biomarkers for preoperative typing in FCD, aiding in surgical decision-making. Future research could involve more FCD pathological subtypes to more comprehensively explore the biological markers of FCD, expand the sample size, and evaluate the predictive efficacy, enhancing its feasibility as a clinical diagnostic tool. Combining the sequencing results from EVs and brain tissue, the study matched the identified DEMs with DEGs in brain tissue and conducted gene function enrichment analysis, selecting functionally distinct cellular pathways. Future studies could target these cellular pathways and target genes for functional validation, aiding in further understanding the pathogenesis and epileptogenic mechanisms of FCD and exploring new targets for drug treatment.

## Supplementary information

Supplementary Figure

Supplementary Table S1

Supplementary Table S2

Supplementary Table S3

Supplementary Table S4

Supplementary Table S5

Supplementary Table S6

Supplementary Table S7

## Data Availability

The raw sequence data reported in this paper have been deposited in the Genome Sequence Archive^49^ in the National Genomics Data Center,^50^ China National Center for Bioinformation/Beijing Institute of Genomics, Chinese Academy of Sciences (GSA: HRA008236), which are publicly accessible at https://ngdc.cncb.ac.cn/gsa.
