# Supplementary material for: Plasma extracellular vesicles-derived microRNAs provide potential biomarkers in distinguishing between focal cortical dysplasia type I and II
Source: Pediatr Res. 2025 Nov 2;100(3):1421–31. doi: 10.1038/s41390-025-04343-z (PMC13672060; doi:10.1038/s41390-025-04343-z)
Supplement: Supplementary file 1 [file 41390_2025_4343_MOESM1_ESM.pdf]

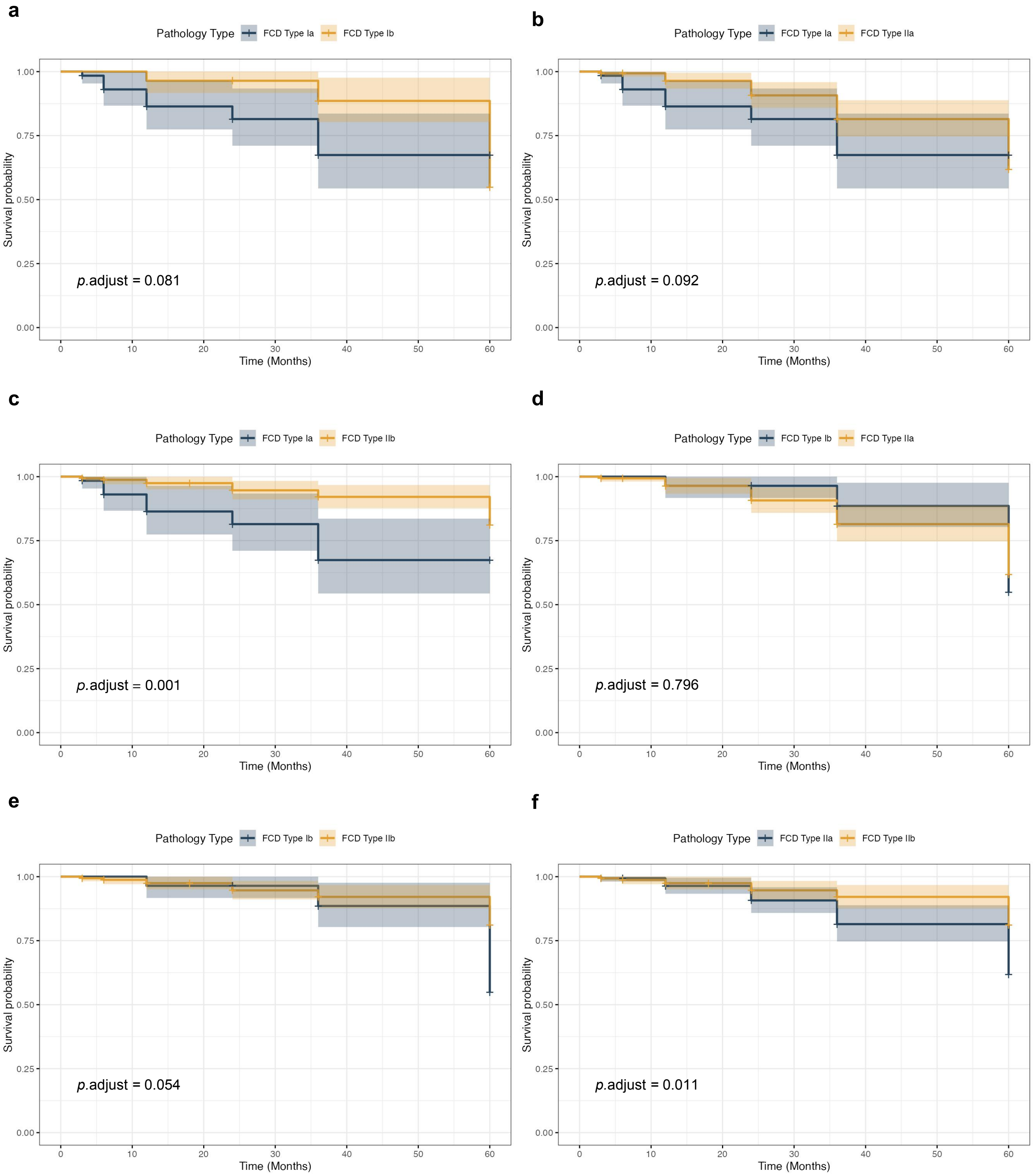

Supplementary Figure S1. Comparison of surgical prognosis among 4 pathological subtypes of FCD. **(a)** The KM curves of surgical prognosis for FCD type Ia (seizure free rate = 77.78%) and Ib (seizure free rate = 75.00%). There is no statistically significant difference. **(b)** The KM curves of surgical prognosis for FCD type Ia and IIa (seizure free rate = 77.42%). There is no statistically significant difference. **(c)** The KM curves of surgical prognosis for FCD Ia and IIb (seizure free rate = 77.42%). There is a statistically significant difference between the two pathological subtypes (*p*.adjust < 0.05). **(d)** The KM curves of surgical prognosis for FCD Ib and IIa. There is no statistically significant difference. **(e)** The KM curves of surgical prognosis for FCD Ib and IIb. There is no statistically significant difference. **(f)** The KM curves of surgical prognosis for FCD IIa and IIb. There is a statistical difference between the two pathological subtypes (*p*.adjust < 0.05). Multiple comparisons were performed using the Benjamini-Hochberg method (BH).

Abbreviations: FCD, focal cortical dysplasia; KM curves, Kaplan-Meier curve.

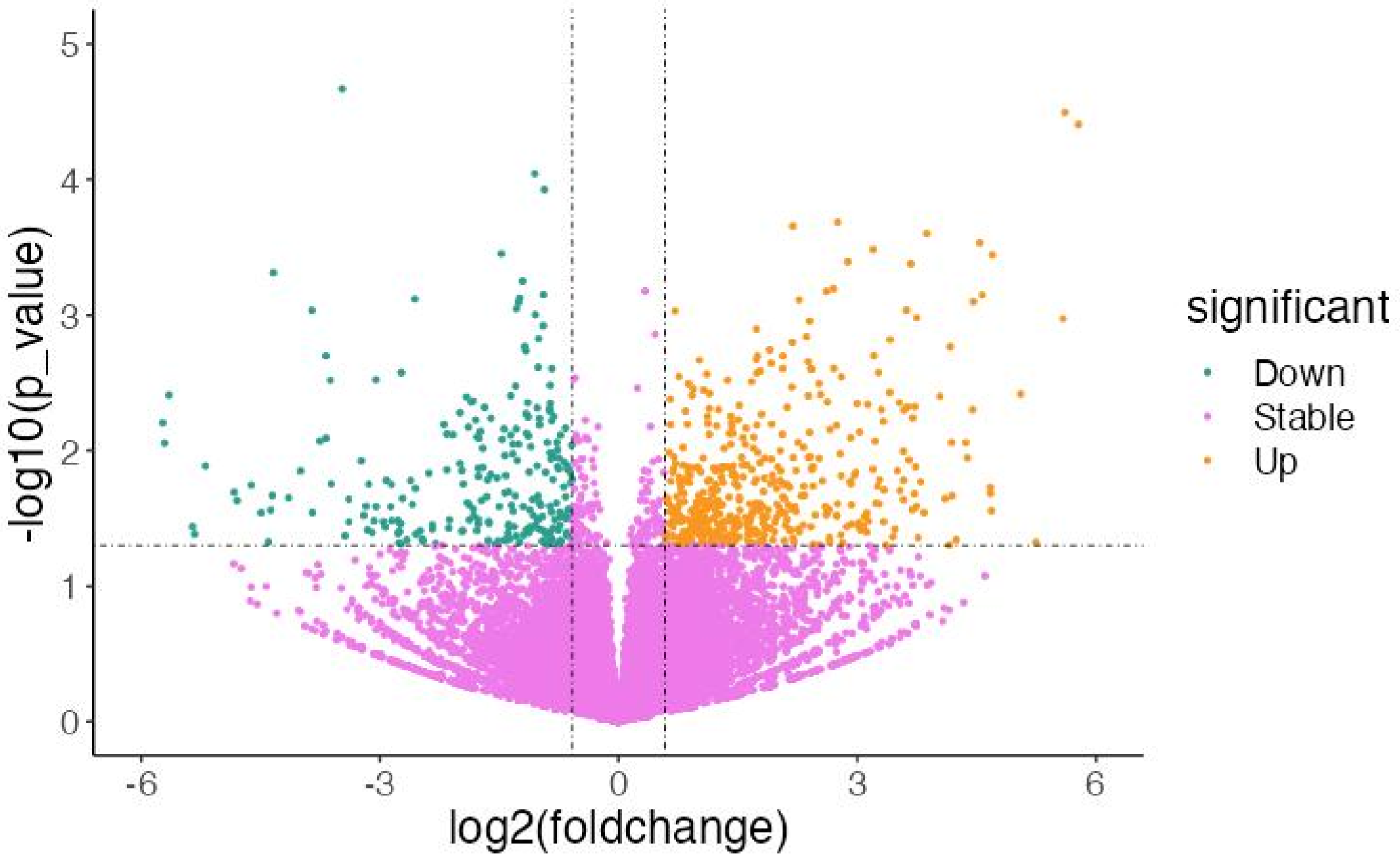

Supplementary Figure S2. The volcano plot of gene expression differences between FCD Ia and IIb brain tissues. Genes that were up-regulated in FCD IIb compared to Ia were shown in yellow, whereas genes that were down-regulated in FCD IIb relative to Ia were marked in green. Stable miRNAs were not differentially expressed between FCD IIb and Ia and were labeled pink on the graph. Compared to FCD Ia, 422 genes were up-regulated, and 161 genes were down-regulated in FCD IIb.

Abbreviations: FCD, focal cortical dysplasia
